# Expressions of TIMP-1, COX-2 and MMP-7 in Colon Polyp and Colon Cancer

**DOI:** 10.5005/jp-journals-10018-1138

**Published:** 2016-07-09

**Authors:** Gösel Bengi, Didem Keles, Ömer Topalak, Mustafa Yalçin, Rabia Kiyak, Gülgün Oktay

**Affiliations:** 1 Department of Gastroenterology, Dokuz Eylul University Hospital, izmir, Turkey; 2 Department of Biochemistry, Dokuz Eylul University Hospital, izmir, Turkey

**Keywords:** Adenomatous polyposis of the colon, Colonic neoplasms, Cyclooxygenase-2, Matrix metalloproteinase-7, Tissue inhibitor of metalloproteinase-1.

## Abstract

**Objective:**

We aimed to investigate the relationship of expression of matrix metalloproteinase-7 (MMP-7), tissue inhibitor of metalloproteinase-1 (TIMP-1) and cyclooxygenase-2 (COX-2) in colon cancer and its predecessor colon polyp.

**Materials and methods:**

This study included 29 patients with colon polyp, 19 patients with colon cancer and 65 healthy control subjects. The expressions of MMP-7, TIMP-1 and COX-2 were investigated by real time-polymerase chain reaction (RT-PCR).

**Results:**

The expressions of TIMP-1, COX-2 and MMP-7 levels were significantly higher in polyp tissue compared to normal tissue (p = 0.024, p < 0.001, p = 0.009, respectively). Expression of TIMP-1, COX-2 and MMP-7 in cancer tissues were higher than both normal tissue and polyp tissue (p = 0.009 and p = 0.001; p < 0.001 and p < 0.001; p = 0.029 and p = 0.008, respectively). In the cancer group, no significant relationship was detected between metastasis and MMP-7, TIMP-1 and COX-2 expressions (p > 0.05). In the polyp tissues, no significant relationship was detected between the histologic type and size of polyps and MMP-7, TIMP-1 and COX-2 levels (p > 0.05). The areas under the receiver operating characteristic (ROC) curve for the cancer group were 0.821 for TIMP-1, 0.888 for COX-2, and 0.880 for MMP-7 (p = 0 < 0.001).

**Conclusion:**

A role and implication of expressions of MMP-7, COX-2 and TIMP-1 in colon cancer is predicted.

**How to cite this article:**

Bengi G, Keles D, Topalak Ö, Yalçin M, Kiyak R, Oktay G. Expressions of TIMP-1, COX-2 and MMP-7 in Colon Polyp and Colon Cancer. Euroasian J Hepato-Gastroenterol 2015;5(2):74-79.

## INTRODUCTION

Colorectal cancer (CRC) is one of the most common malignancies in developed countries. The vast majority of CRC (98%) are adenocarcinomas, which almost always grow from adenomatous polyps. Colorectal cancer development is a multi-step process that occurs as a result of mutations in oncogenes, such as K-ras, adenomatous polyposis coli (APC) gene and tumor suppressor p53 gene that lead to cellular degeneration and uncontrolled cell proliferation. Early diagnosis is important in CRC because the cure rates are higher in patients without metastasis. Colonoscopy and sigmoidoscopy are highly specific and sensitive tests used in detecting colon cancer; however, they are invasive procedures and the efficiency of the procedure varies based on both the patient’s compliance and the experience of the physician performing the procedure. Although the fecal occult blood test (FOBT) is a noninvasive and simple test, less than 10% of positive results are truly CRC positive. When all these tests are considered, serum biomarkers are still not diagnostic for CRC.

Extracellular matrix (ECM) is a structure that supports the cells and communication between cells. The destruction of ECM and basement membrane is an essential step in tumor invasion and metastasis. Proteolytic enzymes control the catabolism of ECM. Matrix metalloproteinases (MMPs) are zinc-dependent endopeptidase family proteins that break down the ECM and basement membrane components.^1^ The disturbance of balance between MMPs and tissue inhibitor of metalloproteinases (TIMPs) is important in tumor invasion and metastasis. Matrix metalloproteinase-7 (MMP-7) is also known as matrilysin and plays a role in apoptosis, angiogenesis, tumor growth, invasion and metastasis.^2^ In addition, MMP-7 activates other metalloproteinases, such as MMP-2 and MMP-9 and these may affect ECM and causes the invasion of cancer cells. Physiologically, MMP-7 is also found in ductal and glandular epithelium of many tissues, as well as in monocytes and mesenchymal cells.^3^ Matrix metalloproteinase-7 has been shown to be important in colon adenoma and in the development of colon cancer and metastasis.^4,5^

Tissue inhibitor of metalloproteinase-1 (TIMP-1) is an inhibitor of many MMPs. Experimental studies revealed that, while TIMP-1 levels were within normal reference values in healthy donors, they were found to be significantly higher in patients with CRC.^6^ Tissue inhibitor of metalloproteinases exhibit complex dual effects on tumor progression, on one hand directly inhibiting MMPs and on the other hand affecting angiogenesis, inhibiting apop-tosis of tumor cells and therefore leading to metastasis of tumor cells. The correlation between TIMP-1 mRNA overexpression and CRC progression and TIMP seems to have a prognostic importance.^7,8^

Cellular proliferation is the basis of tumor development and cyclooxygenases (COXs) are one of the main enzymes that regulate it. Cyclooxygenase plays an important role in the development of metaplastic and dysplastic tissue as well as in development and progression of cancer. Moreover, while COX-2 is expressed in 80 to 90% of CRC cases and in 40 to 50% of premalignant adenomas, it is not expressed in the normal colon tissue. This indicates that COX-2 might have a role in colorectal carcinogenesis.^6^ It has been observed that tumors progress more aggressively in CRC patients with elevated COX-2.^9^

The aims of this study were to compare tissue levels of TIMP-1, COX-2 and MMP-7 in cancer, polyps and control groups to reveal their impact in carcinogenesis and metastasis.

## MATERIALS AND METHODS

### Patients and Tissue Samples

The study was initiated after clinical and laboratory research ethics committee approved the study. The informed consents were obtained from all patients. Patients with inflammatory bowel diseases and patients that received aspirin and nonsteroidal anti-inflammatory drugs (NSAIDs) in the previous 3 days were excluded from the study. This study included patients that had undergone lower gastrointestinal endoscopy and were determined to have colon polyps that were biopsied (colon polyp group, n = 29) and colon cancer (colon cancer group, n = 19) and patients that underwent colonoscopy for any reason and whose normal mucosa sample was obtained for screening purposes (healthy population group, n = 65). Tissue samples obtained from patients were at least 5 mm in size and were used to investigate the expression of MMP-7, TIMP-1 and COX-2 by (RT-PCR). The clinic-pathological data of patients were obtained from the hospital information systems.

### Total RNA Isolation and Real Time Polymerase Chain Reaction

Total rebonucleic acid (RNA) was extracted from tumor, polyp and normal colorectal biopsy samples using Trizol reagent (Roche Applied Science, Indianapolis, IN, USA) and reverse transcribed to obtain complementary DNA (cDNA) with First Strand cDNA synthesis kit (Roche Applied Science, Indianapolis, IN, USA), respectively, in accordance with the manufacturer’s instructions. PCR primers and Taqman hydrolysis probes for MMP-7, COX-2, TIMP-1 and β-actin genes were obtained from Roche Applied Science (Roche Applied Science, Indianapolis, IN, USA). β-actin was used as a housekeeping gene. Real Time PCR was performed using Light Cycler 480 Probes Master kit (Roche Applied Science, Indianapolis, IN, USA), which is a ready-to-use reaction mix that contains FastStart Taq DNA Polymerase, under the following conditions: an initial denaturation for 10 minutes at 95°C, 40 cycles of denaturation for 10 seconds at 95°C, annealing for 30 seconds at 60°C and elongation for 10 seconds at 72°C. LightCycler 480 (V1.5.0) Software (Roche Applied Science, Indianapolis, IN, USA) was used to measure the CT values and relative mRNA expression levels were calculated with comparative 2-AACT method.

## STATISTICAL ANALYSIS

Statistical package for the social sciences (SPSS) 15.0 software (SPSS Inc, Chicago, IL, USA) was used for statistical analyses. Nonparametric Kruskal-Wallis test was used for the comparison of means between independent groups. Nonparametric Mann-Whitney U test was used for comparing paired subgroups. The relationship between clinicopathological data and tissue TIMP-1, MMP-7 and COX-2 was analyzed using the non-parametric Mann-Whitney U test. Spearman correlation test was used to determine the relationship between polyp size and tissue TIMP-1, MMP-7 and COX-2. The diagnostic value of tissue TIMP-1, MMP-7 and COX-2 was evaluated by the receiver operating characteristic (ROC) curve as the area under the curve (AUC). The optimal cut-off levels were calculated based on the Yoden index, where sensitivity and specificity were assumed to be equally important. Logistic regression method was used for multivariate analysis. The confidence level of 95% was employed and p < 0.05 was considered statistically significant.

## RESULTS

There was no significant difference between the control, polyp and cancer groups in terms of age and gender (p = 0.73 for age and p = 0.87 for gender) (Table 1). The mean TIMP-1, COX-2 and MMP-7 levels were significantly higher in polyp tissue compared to normal tissue (p = 0.024, p < 0.001, p = 0.009, respectively). The levels of TIMP-1, COX-2 and MMP-7 in cancer tissues were higher than those of both normal tissue and polyp tissue (p = 0.009 and p = 0.001; p < 0.001 and p < 0.001; p = 0.029 and p = 0.008, respectively) (Tables 2 to 4).

There was no significant relationship between MMP-7, TIMP-1 and COX-2 levels and presence or absence of metastasis in the cancer group (Table 5). In the polyp group, no significant relationships between MMP-7, TIMP-1 and COX-2 levels and the histologic types of polyps were detected (Table 6). When analysis performed using the Spearman correlation test revealed that there was no significant relationship between TIMP-1, COX-2 and MMP-7 levels and polyp size (Table 7). This evaluation included polyp size being smaller or larger than 6 mm.

**Table 1:** **Table 1:** Descriptive statistics

*Tissue*		*(n)*		*Percentage*	
Normal		65		57.5	
Polyp		29		25.7	
Cancer		19		16.8	
Total		113		100	
*Metastasis (n, %)*					
Yes		4		21.1	
No		15		78.9	
Total		19		100	
*Polyp Type (n, %)*					
Tubular		24		82.8	
Tubulovillous + Villous		5		17.2	
Total		29		100	
Polyp size (Mean, SD)		12.62		6.76	

SD: Standard Deviation

**Table 2:** **Table 2:** Comparison of TIMP-1 levels in control, polyp and cancer groups

		*N*		*Minimum*		*Maximum*		*Mean*		*SD*		*p*	
Control		65		0.00		173.95		42.56		40.90		0.024	
Polyp		29		0.00		620.60		97.62		126.86			
Control		65		0.00		173.95		42.56		40.90		0.001	
Cancer		19		21.29		918.79		154.18		204.74			
Polyp		29		0.00		620.60		97.62		126.86		0.009	
Cancer		19		21.29		918.79		154.18		204.74			

TIMP-1: Tissue inhibitor of metalloproteinase-1; SD: Standard Deviation

**Graph 1:**
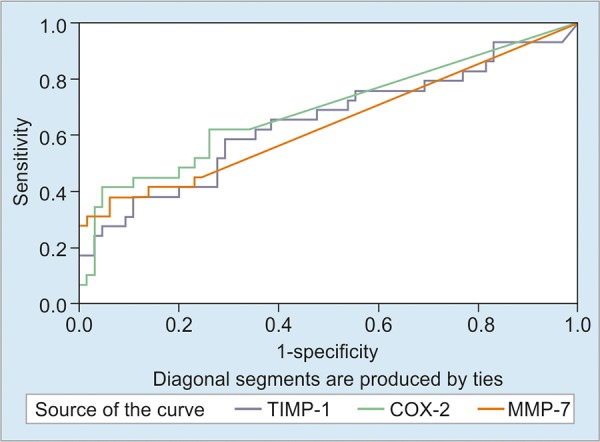
Area under the ROC curve for the polyp group

**Graph 2:**
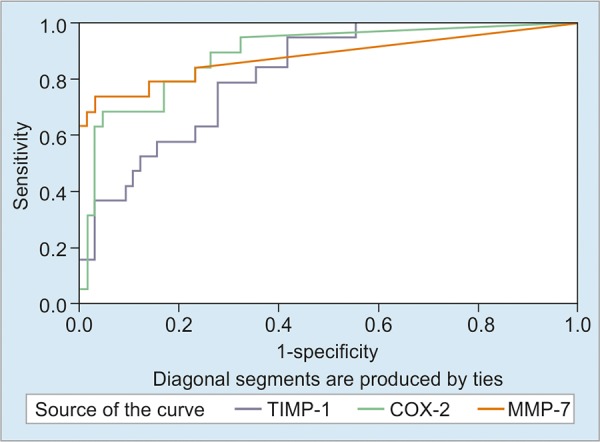
Areas under the ROC curves for the cancer group

The areas under the curve for TIMP-1, COX-2 and MMP-7 were determined to be 0.647, 0.689 and 0.639, respectively and were found to be statistically significant (p = 0.024, p = 0.003 and p = 0.032, respectively) in the polyp group (Graph 1). Also the areas under the curve were 0.821 for TIMP-1, 0.888 for COX-2 and 0.880 for MMP-7 statistically significant (p = 0 < 0.001) in the cancer group (Graph 2). The cut-off values were calculated using the Youden Index, and the sensitivity and specificity ratios of these values have been shown in Tables 8 and 9.

The cut-off values of the markers in polyp and cancer tissues were evaluated with two different logistic regression equations. The cut-off value for COX-2 was 17, and the probability of developing cancer was 15 times more likely in values above 17 compared to values below 17 (1.5-142.4). The cut-off value for MMP-7 was 33.62 and the probability of developing cancer was 80 times higher in values above 33.62 compared to the values below 33.62 (10.1-635.7) (Table 10).

**Table 3:** **Table 3:** Comparison of COX-2 levels in control, polyp and cancer groups

		*N*		*Minimum*		*Maximum*		*Mean*		*SD*		*p*	
Control		65		0.00		143.74		5.59		19.22		<0.001	
Polyp		29		0.00		611.86		39.87		114.18			
Control		65		0.00		143.74		5.59		19.22		<0.001	
Cancer		19		0.00		196.72		51.40		57.90			
Polyp		29		0.00		611.86		39.87		114.18		<0.001	
Cancer		19		0.00		196.72		51.40		57.90			

COX-2: Cyclooxygenase-2; SD: Standard deviation

**Table 4:** **Table 4:** Comparison of MMP-7 levels in control, polyp and cancer groups

		*N*		*Minimum*		*Maximum*		*Mean*		*SD*		*p*	
Control		65		0.00		52.75		4.99		11.23		0.009	
Polyp		29		0.00		10962.35		470.34		2031.79			
Control		65		0.00		52.75		4.99		11.23		0.029	
Cancer		19		0.00		11736.31		856.33		2652.81			
Polyp		29		0.00		10962.35		470.34		2031.79		0.008	
Cancer		19		0.00		11736.31		856.33		2652.81			

MMP-7: Matrix metalloproteinase-7; SD: Standard deviation

**Table 5:** **Table 5:** Comparison of tissue TIMP-1, MMP-7 and COX-2 levels according to clinicopathological parameters in the cancer group

		*MET*		*N*		*Mean*		*SD*		*p*	
TIMP-1		No		15		172.88		226.63		0.549	
		Yes		4		84.03		59.32			
COX-2		No		15		38.91		45.07		0.110	
		Yes		4		98.27		83.19			
MMP-7		No		15		1036.93		2978.43		0.483	
		Yes		4		179.10		227.06			

TIMP-1: Tissue inhibitor of metalloproteinase-1; COX-2: Cyclooxygenase-2; MMP-7: Matrix metalloproteinase-7; SD: Standard deviation

**Table 6:** **Table 6:** Comparison of plasma TIMP-1, MMP-7 and COX-2 levels according to clinicopathological parameters (polyp type) in the polyp group

		*Polyp type*		*N*		*Mean*		*SD*		*P*	
TIMP-1		Tubular		24		110.32		136.15		0.236	
		Tubulovillous + Villous		5		36.70		21.73			
COX-2		Tubular		24		43.75		125.11		0.905	
		Tubulovillous + Villous		5		21.27		27.10			
MMP-7		Tubular		24		504.16		2229.64		0.311	
		Tubulovillous + Villous		5		308.01		522.11			

TIMP-1: Tissue inhibitor of metalloproteinase-1; COX-2: Cyclooxy-genase-2; MMP-7: Matrix metalloproteinase-7; SD: Standard deviation

**Table 7:** **Table 7:** Comparison of plasma TIMP-1, MMP-7, COX-2 levels according to clinicopathological parameters (polyp size) in the polyp group

		*Polyp size*			
		*r*		*P*	
TIMP-1		0.246		0.199	
COX-2		–0.012		0.949	
MMP-7		0.062		0.749	

TIMP-1: Tissue inhibitor of metalloproteinase-1; COX-2: Cyclooxy-genase 2; MMP-7: Matrix metalloproteinase 7; SD: Standard deviation

**Table 8:** **Table 8:** Areas under the ROC curves, cut-off values and sensitivity-specificity rates for TIMP-1, COX-2 and MMP-7 in the polyp group

		*Area*		*P*		*cut-off*		*Sens*		*Speci*	
TIMP-1		0.647		0.024		54.61		0.586		0.708	
COX-2		0.689		0.003		17.29		0.414		0.954	
MMP-7		0.639		0.032		26.10		0.379		0.938	

Sens: sensitivity; Speci: specificity; ROC: Receiver operating characteristic; TIMP-1: Tissue inhibitor of metalloproteinase-1; COX-2: Cyclooxygenase-2; MMP-7: Matrix metalloproteinase-7; SD: Standard deviation

**Table 9:** **Table 9:** Areas under the ROC curves, cut-off values, and sensitivity-specificity rates for TIMP-1, COX-2 and MMP-7 in the cancer group

		*Area*		*p*		*Cut-off*		*Sen*		*Specificity*	
TIMP-1		0.821		<0.001		36.86		0.947		0.585	
COX-2		0.888		<0.001		17		0.684		0.954	
MMP-7		0.880		<0.001		33.62		0.737		0.969	

Sen: sensitivity; ROC: Receiver operating characteristic; TIMP-1 Tissue inhibitor of metalloproteinase-1; COX-2: Cyclooxygenase-2 MMP-7: Matrix metalloproteinase-7; SD: Standard deviation

## DISCUSSION

The CRC formation, takes place with the conversion of normal epithelial cells to cancer cells, is a complex, multi-step, long process that results in genetic and phe-notypic diversity. Mutations in some tumor suppressor genes and proto-oncogenes, such as deleted in colorectal carcinoma (DCC), p53 and K-ras may have critical role in CRC pathogenesis. Also, imbalance between MMPs and TIMPs may be an important factor in the development of gastrointestinal malignancies.^10,11^ We used quantitative RT-PCR, which is the most sensitive method for the assessment of specific mRNA, for detection of differences in the expression of genes between tumor tissue and control tissue.

**Table 10:** **Table 10:** Logistic regression analysis

								*95% CI for EXP (B)*			
		*B*		*Sig.*		*OR*		*Lower*		*Upper*	
CaCOX-2		2.699		0.019		14.864		1.551		142.441	
CaMMP-7		4.386		<0.001		80.279		10.138		635.708	
PlpCOX-2		2.635		<0.001		13.939		3.277		59.292	
PlpMMP-7		2.179		0.002		8.838		2.25		34.711	

Sig: significant; OR: Odds ratio; CI: Confident interval; TIMP-1: Tissue inhibitor of metalloproteinase-1; COX-2: Cyclooxygenase-2; MMP-7: Matrix metalloproteinase-7

In one study, MMP-7 was shown to be focally over-expressed in about 50% of benign adenomas,^12^ while another study reported that MMP-7 expression was higher in areas of tumor that were more dysplastic and invasive.^13^ Moreover, the overexpression of MMP-7 was observed in all cases with familial adenomatous polyposis regardless of the polyp size and the degree of dysplasia.^14^ However, there is lack of consensus about this. While Luo et al found that in cancer patients MMP-7 expression was correlated with stage, age and lymph node metastasis,^15^ Gomes et al reported no correlation between MMP-7 levels and clinicopathologic variables.^16^ In the present study, we did not find any significant differences between early and stages of the disease in terms of tissue MMP-7 levels. Maurel et al reported that elevated levels of MMP-7 in serum might be evaluated as signs for poor prognosis in patients with advanced CRC.^17^ Wang et al showed that there is a link between MMP-7 expression and the depth of invasion of the intestine wall by the tumor and the presence of distant metastases.^18^ Here, we also determined that there was a significant difference between the mean MMP-7 expression of control, polyp and cancer groups, and that MMP-7 expression increased in polyp and cancer tissue compared to normal tissue (p < 0.001 for both).

During tumor progression, the increased secretion of MMPs from tumor cells or from tumor-related fibroblasts is observed and can be inhibited by TIMP. During this step, more MMP is secreted, and this increases the local TIMP secretion. When the balance between MMP and TIMP is disrupted, restructuring of ECM occurs. Furthermore, tumor progression leads to local tissue hypoxia, and thus the secretion of MMP-7 mediated angiogenic factors is increased. Tissue inhibitor of metalloproteinases-1 protein expression was detected in stromal and epithelial cells of both colonic polyps (n = 29) and adenocarcinoma (n = 25). In this study, the intensity of staining increased from hyperplastic polyps to tubulovillous adenoma and adenocarcinoma.^19^ The increasing serum antigen concentrations of MMPs and TIMPs coincide with a multistep process of colonic carcinogenesis. In this study, we found a significant difference between control, polyp and cancer groups in terms of expression of TIMP-1 (p < 0.001). The diagnostic value of TIMP-1 in distinguishing between polyps and cancer was determined by ROC curve. Our results are in agreement to the findings of Mroczko et al^20^ who revealed that serum concentrations of MMP-9 and TIMP-1 were significantly higher in adenoma patients compared to control group, but lower than in patients with CRC.

Tissue inhibitor of metalloproteinases were suggested to predict the response to chemotherapy. Tissue inhibitor of metalloproteinases-1 is important in determining progression-free survival in metastatic CRC patients receiving combination chemotherapy and showed that increased marker levels were correlated with poor prognosis.^21^ Also, it has been shown that TIMP-1 can be used as an additional marker in evaluation of the chemotherapy response.^22^ In recent years, many synthetic MMPIs have been studied in phase III clinical trials. However, many agents were not successful in terms of efficacy and side effects, so to date there is no therapeutic agent that has been introduced to routine use.

Studies have shown elevated COX-2 expression in CRC and colorectal adenomas compared to normal tissues.^23-25^ A recent study revealed that fecal COX-2 and MMP-7 mRNA levels were elevated in patients with CRC, and that fecal RNA tests were positive in 93% of stages I or II patients.^26^

Taken together, we propose that detection of fecal COX-2 and MMP-7 mRNA level could be considered as a potential test for CRC screening but cautions should be taken to explain the role these variables and more studies would be required to affirm these facts in independent manner.
